# Clinical and pathological characteristics of extra-cranial germ cell tumors: A 30-year single-center experience in Saudi Arabia

**DOI:** 10.15537/smj.2023.44.5.20230070

**Published:** 2023-05

**Authors:** Dania A. Monagel, Aletani Tala, Alghamdi Arwa, Banjar Sereen, Hakeem Ilana, Hannef Deena, Ahmed Omaima, Elimam Naglla

**Affiliations:** *From the College of Medicine (Monagel, Tala, Arwa, Sereen, Ilana), King Saud bin Abdulaziz University for Health Sciences; from the Department of Medical Researches (Monagel), King Abdullah International Medical Research Center; from the Department of Oncology (Monagel, Omaima, Naglla), Ministry of the National Guard- Health Affairs; from the Center of Excellence in Genomic Medicine Research (Deena), King Abdulaziz University, Jeddah, Kingdom of Saudi Arabia.*

**Keywords:** germ cell tumor, pediatric, childhood

## Abstract

**Objectives:**

To investigate the clinical and pathological characteristics of extracranial germ cell tumors (GCTs) in children aged 0–168 months treated at the National Guard Hospital, Jeddah, Saudi Arabia from 1990 to 2020.

**Methods:**

In this retrospective analysis, the data for all cases of GCTs were collected from 1990 to 2020. Statistical analyses were carried out using JMP software. The data was divided into 4 main categories: demographics, pathological/clinical features, recurrence, treatment and outcome.

**Results:**

The study included 50 patients, with a mean age at diagnosis of 56.52 months. The median follow-up duration was 30 months. Most tumors were in the gonads, and among the extragonadal tumors, the sacro-coccyx was the most frequent site of the disease.

The most common histological subtype of GCTs is yolk sac tumor, accounting for 28% of cases. Of the 50 patients, 46% received chemotherapy, and 54% underwent surgery without chemotherapy. Ten (20%) patients experienced recurrence after treatment. At the last follow-up, 96% of the patients were alive, and only 2 of the patients died due to advanced disease.

**Conclusion:**

Our findings were comparable to international data, but improvement in surveillance is required for long-term survivors.

**E**xtracranial germ cell tumors (GCTs) originate from primordial germ cells in the gonads.^1^ Generally, the classification of GCTs is based on the tumor’s location, into gonadal and extra-gonadal.^2^ The most common primary sites of extra-gonadal tumors are the sacrococcygeal, retroperitoneum, mediastinum, head, and neck.^2^ Germ cell tumors may also be classified histologically by the type of tissue in which the cancer cell originates, such as germinomatous (GGCT) and seminomatous (SGCT).^3^ Germ cell tumors can have either benign or malignant behaviors, with the latter being able to be categorized broadly into teratoma, mixed GCTs, or malignant GCTs.^1^

Germ cell tumors are rare childhood malignancies accounting for only 4% of all pediatric cancers.^3^ The prevalence of GCTs is approximately 8-10 cases per 100,000 across all age groups.^4,5^ Naturally, GCTs have a bimodal age distribution with the first peak in children aged 0-4 years, following by the age ranging from puberty until early adulthood.^6^ In Saudi Arabia (1994-2012), the incidence rates of GCTs have been reported to be 4.5 million per year among children 0-19 years of age.^7^

Specific syndromes/abnormalities are associated with GCTs, namely, Klinefelter, Swyer, Turner, and Down syndromes, as well as cryptorchidism, and gonadal dysgenesis.^8^ The Children Oncology Group (COG) reported worse outcomes among patients with ovarian non-dysgerminomatous tumors if accompanied by gonadal dysgenesis.^9^ The same group had recommended screening for Klinefelter syndrome in patients with mediastinal GCTs, as 1/3 of men with mediastinal GCTs had Klinefelter syndrome in their cohort.^10^

Since GCTs are histologically diverse, the treatment choice primarily depends on the tumor’s histology and tumor markers, such as α-1-fetoprotein (AFP) and beta human chorionic gonadotropin (bHCG).^2^ In extracranial GCTs, treatment options vary between surgical resection/biopsy followed by observation or chemotherapy, depending on the histology and degree of resection. Different combinations of chemotherapy regimens are used with comparable outcomes, but primarily platinum-based chemotherapy has been utilized.^11,12^ Additionally, GCTs respond well to radiotherapy; however, they are rarely used because of their long-term side effects.^13^

Germ cell tumors are rare pediatric cancers with excellent outcomes when treated according to well-established international guidelines.^14^ Generally, the outcome of pediatric patients with extracranial GCTs is exceptional depending on the tumor site, histology, stage, and treatment used.^11,12^ International studies reported an overall survival rate of 90-95% for low risk and approximately 85% for malignant GCTs.^14^ In Saudi Arabia, a study that has been carried out reported an overall survival rate of approximately 95% at 35 months among children with testicular GCTs.^15^

Williams et al^16^ showed disparities between survival and ethnicity across a different group of children with GCTs, which may be affected by variations in the exposures, tumor-related biology, or treatment administered. In Saudi Arabia, there is a paucity of reports addressing the epidemiology, clinical characteristics, and outcomes of pediatric patients with GCTs. Therefore, our research aimed to describe the clinical data, treatment modalities, and outcomes of pediatric patients with GCTs who had been treated at a single oncology center in Saudi Arabia for over 30 years.

## Methods

This study was a retrospective descriptive cohort in which charts of all children with extracranial GCTs treated at the National Guard Hospital (NGH) from 1990 to 2020 were analyzed and reviewed. Our study was approved by the Institutional Review Board of King Abdullah International Research Center, Jeddah, Saudi Arabia with a waiver of informed consent. This study was carried out at the Pediatric Oncology Department, National Guard Hospital, Jeddah, Saudi Arabia. The Pediatric Oncology Department is part of the Princess Nora Oncology Center and is considered one of a leading pediatric oncology hospital in the region. On average, 150-200 new pediatric oncology cases are treated annually.

Our research team reviewed 52 charts of patients with extracranial GCTs who attended the NGH Pediatric Oncology Department from January 1990 to January 2020. All children aged from birth to 14 years with a diagnosis of GCTs were included in the study (n=50). In contrast, subjects with intracranial GCTs or those who were lost to follow-up before the completion of the treatment protocol were excluded.

Data collected included demographic variables, GCTs diagnosis-related information, such as age at diagnosis, symptoms, tumor site, tumor markers, locoregional extension, tumor size, presence of metastatic disease, lymph node involvement, histological classification, and risk stratification. Other data of interest included treatment modality, survival at the last follow-up, long-term effects (such as hearing/renal impairments and pulmonary toxicity), and disease recurrence.

Tumors were categorized into gonadal and extragonadal GCTs. The histological characteristics were categorized according to modified Denher’s classification into germinoma, teratoma, embryonal carcinoma, yolk sac tumor, choriocarcinoma, gonadoblastoma, mixed GCTs, and others.^1^ The risk stratification (low, intermediate, or high-risk tumors) was followed as per the United Kingdom Children’s Cancer Study Group Protocol, Germ Cell Working Group (UKCCSG GCIII ‘GC 2005 04’).^17^ Staging for malignant GCTs at all sites was based on tumor, node, and metastasis (TNM) staging system.^18^

### Statistical analysis

Data analysis was carried out using the John’s Macintosh Project software, version 10.0 (SAS Institute Inc., Cary, NC, USA). Parametric approaches using means, medians, ranges, and standard deviations (SD) were used to describe the numerical data. Frequency, proportions, and bar graphs were used to describe the categorical variables. Survival probability was analyzed using the Kaplan-Meier method, with censoring as needed based on the duration of follow-up.

## Results

The study included 50 pediatric patients aged 0–14 years who were diagnosed with extracranial GCTs over 30 years (1990-2020). The mean age at the time of diagnosis was 4.71 years (SD, 4.75). GCTs were more common in female patients than in male patients at a ratio of 2:1, and the mean age at diagnosis was higher in girls at 6.6 years (SD, 4.39) while 1.4 years (SD, 1.4) in boys.

A total of 66% of the tumors were in the gonads, compared to 34% which were in the extragonadal sites. Of the gonadal tumors, 48.5% were in the testes, and 51.5% were in the ovaries. Among the extragonadal tumors, the sacro-coccyx was the most prevalent, constituting approximately 52.9% of cases. Other extragonadal sites included the abdomen “liver, abdominal wall” (17.7%), retroperitoneum (11.8%), mediastinum (11.8%), and intraorbital (5.9%).

The most common symptom at initial presentation was related to the abdomen (pain/distension) in approximately 50% of patients. Furthermore, abdominal symptoms were more commonly associated with ovarian GCT than with other tumor sites. The third most common presenting symptom was testicular swelling, which was found in 18% of patients. Overall, the patients with ovarian GCT had the highest number of symptoms reported at presentation. In addition, AFP was elevated in 73% and bHCG levels elevated in 14% of patients.

Regarding tumor size, 35.4% of patients had a tumor size of less than 5 cm and 64.6% had a tumor size equal to or larger than 5 cm. The log-rank test showed no statistically significant difference in the rate of recurrence between the 2 groups (*p*=0.63).

Positive lymph node involvement was observed in 32% of the patients, and 29% had positive locoregional extension. Eight (17.4%) patients had distant metastasis. The 2 most common sites of distant metastasis were the lungs (n=2) and brain (n=2), followed by the peritoneum, liver, bone, and pleura (n=1).

The risk group was divided into high-(26.5%), intermediate-(38.8%), or low-(34.7%) risk. The low-risk group was more frequent in boys and the intermediate-risk/high-risk groups were more common among girls. Tumor stages were recorded in 48 patients, with only 2 subjects in the unknown stage, as summarized in Table 1.

**Table 1:** - Tumor stage in relation to tumor site and tumor markers (AFP, Beta- HCG).

Stage	n (%)	Site	AFP	Beta-HCG
Stage 1	18 (37.5)	◾ Testes: 13 patients ◾ Ovaries: 4 patients ◾ Sacro-coccyx: 1 patient	Normal 5 patients High: 13 patients	Normal: 13 patients High: 1 patient Missing: 4 patients
Stage 2	6 (12.5%)	◾ Testes: 1 patient ◾ Ovaries: 2 patients ◾ Sacro-coccyx: 1 patient ◾ Retroperitoneum: 1 patient ◾ Abdomen: 1 patient	Normal: 2 patients High: 4 patients	Normal: 6 patients
Stage 3	10 (20.8)	◾ Ovaries: 7 patients ◾ Sacro-coccyx: 1 patient ◾ Retroperitoneum: 1 patient ◾ Mediastinum: 1 patient	Normal: 3 patients High: 7 patients	Normal: 7 patients High: 2 patients Missing: 1 patient
Stage 4	14 (29.1%)	◾ Testes: 2 patients ◾ Ovaries: 4 patients ◾ Sacro-coccyx: 4 patients ◾ Abdomen: 2 patients ◾ Mediastinum: 1 patient ◾ Intra-orbital: 1 patient	Normal: 3 patients High: 9 patients Missing: 4 patients	Normal: 8 patients High: 3 patients Missing: 3 patient

AFP: α-1-fetoprotein, HCG: human chorionic gonadotropin

The most common histological subtype of GCTs is yolk sac tumor, which accounts for 28% of the entire cohort (n=14 patients) and most commonly developed in the testes. When examining risk groups with histological subtypes in low-risk patients, yolk sac GCT was the most common type, followed by mature and immature teratomas. However, immature teratomas were the most common in the intermediate-risk group, followed by dysgerminomas, while mixed and yolk sac GCTs were the most common in the high-risk group.

Only 15 (32%) patients with GCTs underwent biopsy at the tumor site before surgical intervention. Surgical intervention was performed in 84% of patients. Out of the 42 patients who had surgery, 14 patients underwent surgical excision of the tumor, 14 of the male patients with testicular GCTs underwent orchiectomy. Of the 14 female patients with ovarian GCT, 7 had an oophorectomy while the remaining 7 had a salpingo-oophorectomy.

Of the 50 patients, 46% received chemotherapy, and 54% underwent surgery without chemotherapy. The local treatment protocol (GCT09) was based UKCCG GCIII (GC 2005 04). Most patients who received chemotherapy received between 4 and 6 cycles. Table 2 summarizes the treatments in relation to stage.

**Table 2:** - Treatment in relation to stage.

Stage	Number of patients	No chemotherapy	Chemotherapy
Stage 1	18	16 (88.9% of stage 1)	2 (11.1% of stage 1)
Stage 2	6	5 (83.3% of stage 2)	1 (16.7% of stage 2)
Stage 3	10	3 (30.0% of stage 3)	7 (70.0% of stage 3)
Stage 4	14	1 (7.1% of stage 4)	13 (92.9% of stage 4)

For patients who received chemotherapy, long-term follow-up was necessary. Tests, such as the pretreatment glomerular filtration rate (GFR) level, post-treatment GFR level, pulmonary function test (PFT), and hearing assessment were performed. However, the pre-GFR and post-GFR levels were only obtained from 9 of the 23 patients who received chemotherapy using nuclear testing Tc-99m diethylene-triamine-pentaacetate, with normal GFR pre- and post-therapy. Hearing assessment was performed in 4 patients and PFT in 3 patients, all of whom had normal results in both tests.

The duration between diagnosis and the last follow-up ranged from 2 to 164 months, with a median duration of follow-up of 30 months. Furthermore, 96% patients are alive, with only 2 of the patients deceased. Both were high-risk, and mortality was related to advanced disease status (uncontrolled bleeding and respiratory compromise).

Approximately 20% of patients experience recurrence after treatment. Of those patients, 4 had local recurrence, 5 had metastasis, and 1 was unknown. Five of the 10 patients with recurrence were treated with surgery only, while the others were treated with both chemotherapy and surgery (2 patients) or unknown treatment (3 patients). When comparing the recurrence to the histological subtype, of the 14 patients with yolk sac tumors, 4 patients experienced recurrence. However, only 1 patient with immature teratoma and 3 patients with mature teratoma experienced recurrence. The remaining 2 patients with recurrence had mixed GCTs.

## Discussion

Pediatric GCTs are rare tumors that include various groups of neoplasms, with variability in histology, sites, clinical presentations, and outcomes. It accounts for 3.5% of all childhood cancers that arise before 15 years of age, although the incidence increases to 13.9% in the adolescent age group, which makes it the most common malignancy in this age group after Hodgkin lymphoma.^14-19^ Since there is a paucity of data in Saudi Arabia regarding this important group of tumors in the pediatric age group, this study aimed to investigate the clinical and pathological characteristics of extracranial GCTs in a single tertiary center over a 30-year period.

Many aspects of our findings were comparable to international data, and GCTs were more frequent among female patients in our cohort. However, girls were older at the time of presentation, which is likely related to gender biological variances.^19^ Incesoy-ozdemir et al^20^ described that abdominal pain and distention were the 2 most common symptoms, which supports our results, although there was a remarkable variation in symptoms at initial presentation based on the site of the tumor. Tumor markers, including AFP and Beta-HCG, were important tools used in the diagnosis, relapse, and follow-up.

Furthermore, the most common histological type in our study was teratoma followed by yolk sac tumor, similar to most published studies on extracranial pediatric GCTs.^21^ The most prevalent tumor site for both genders was the gonads compared to extragonadal sites. Among the extragonadal sites, the sacro-coccyx was the most common, which is consistent to results published by Brodeur et al.^22^ In addition, less than half of the patients had locoregional extension and lymph node involvement, and only 17.4% of the cases had distant metastasis.

Stage 1 disease represents most of our sample, which is probably related to the bimodal age of distribution as characteristics for GCTs and lower stages being more prevalent in younger patients.^21^ Our pediatric team only treats children up to 14 years of age; therefore the second peak during adolescence could not be studied. Patients were treated according to international standards with platinum-based chemotherapy, although less than 50% of the patients received chemotherapy, while the remaining patients only underwent surgery with no chemotherapy.^11^ While GCTs are radiosensitive tumors, none of the patients received radiation as part of the treatment due to late toxicity.^13^

**Figure 1:**
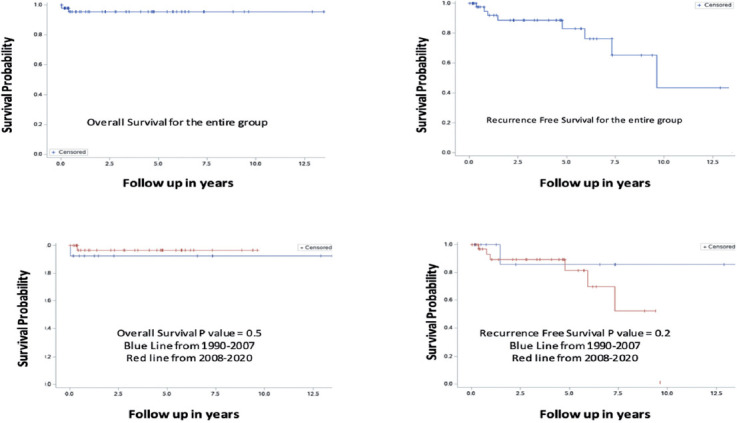
- Kaplan-Meier curves for overall survival and recurrence free survival from 1990-2020.

Figure 1 summarizes the overall survival and recurrence-free survival. Although the individuals in this cohort were treated outside clinical trials, there was good adherence to the international protocol available at the time.^12^ The 5-year overall survival for GCTs range from 91% to 85% with 88% event-free survival, which is comparable to the survival analysis of our group.^12, 23^

Despite these good outcomes, challenges continue. Ototoxicity, nephrotoxicity, neurotoxicity, pulmonary disease, and second malignant neoplasms (SMN) are some of the long-term sequelae associated with long-term survival. Data on late effects were extracted from adult patients who received similar therapy for testicular cancer. Different studies have proven that platinum-related hearing loss might worsen over time, have a late onset, or lead to early onset hearing damage.^14^ GFR can be equally affected as hearing in an adult study estimates at least 15% drops in the GFR, which is irreversible even if the influence is subclinical but can lead to long-term morbidity.^24^

Moreover, there is a rate of 1% of adults treated for testicular cancer per year that accrue SMNs.^25^ Among the same group of patients, there is a 2.5-fold increase in the risk of death from pulmonary disease for individuals who received bleomycin and an 8% prevalence of developing restrictive lung disease.^26,27^ Therefore, these effects can be aggravated in a growing child who receives similar therapy as most of the large late effects studies, such as the childhood cancer survivor study that did not report on GCTs.^28^

### Study limitation

One of the limitations to our study was the lack of long-term outcomes (such as GFR, hearing, lung functions), likely due to the absence of strict protocols and survivor programs to long-term follow up those patients. This calls for a national effort to support the infrastructure of standardizing treatment across our centers in the kingdom, the development of survivorship clinics, and collaboration with the worldwide initiative to cure GCTs with the least possible long-lasting comorbidities.

In conclusion, despite the retrospective nature of the study and its small sample size, this study adds to the growing body of published literature regarding GCTs characteristics among Saudi children. This emphasizes the importance of nationwide protocols and maintaining vigilance screening of long-term survivors.

## References

[1] Dehner LP. Gonadal and extragonadal germ cell neoplasia of childhood. Hum Pathol 1983; 6: 493–511. 10.1016/s0046-8177(83)80004-5 6343221

[2] Göbel U , Schneider DT , Calaminus G , Haas RJ , Schmidt P , Harms D. Germ-cell tumors in childhood and adolescence. GPOH MAKEI and the MAHO study groups. Ann Oncol 2000; 11: 263–271. 10811491 10.1023/a:1008360523160

[3] Khaleghnejad-Tabari A , Mirshemirani A , Rouzrokh M , Mohajerzadeh L , Khaleghnejad-Tabari N , Hasas-Yeganeh S. Pediatric germ cell tumors; A 10-year Experience. Iran J Pediatr 2014; 24: 441–444. 25755868 PMC4339570

[4] Znaor A , Lortet-Tieulent J , Jemal A , Bray F. International variations and trends in testicular cancer incidence and mortality. Eur Urol 2014; 65: 1095–1106. 24268506 10.1016/j.eururo.2013.11.004

[5] Stang A , Bray F , Dieckmann KP , Lortet-Tieulent J , Rusner C. Mortality of testicular cancer in East and West Germany 20 years after reunification: A gap not closed yet. Urol Int 2015; 95: 160–166. 25966659 10.1159/000381883

[6] Frazier AL , Amatruda JF. Pediatric germ cell tumors. Biology treatment survivorship. Anticancer Research 2014; 34: 3234–3235.

[7] Steliarova-Foucher E , Colombet M , Ries LAG , Moreno F , Dolya A , Bray F , et al. International incidence of childhood cancer, 2001-10: a population-based registry study. Lancet Oncol 2017; 18: 719–731. 28410997 10.1016/S1470-2045(17)30186-9 PMC5461370

[8] Dexeus FH , Logothetis CJ , Chong C , Sella A , Ogden S. Genetic abnormalities in men with germ cell tumors. J Urol 1988; 140: 80–84. 2837589 10.1016/s0022-5347(17)41492-3

[9] Dicken BJ , Billmire DF , Krailo M , Xia C , Shaikh F , Cullen JW , et al. Gonadal dysgenesis is associated with worse outcomes in patients with ovarian nondysgerminomatous tumors: a report of the Children’s Oncology Group AGCT 0132 study. Pediatr Blood Cancer 2018; 65: 10.1002/pbc.26913. 10.1002/pbc.26913 PMC6219870 29286555

[10] Nichols CR , Heerema NA , Palmer C , Loehrer PJ, Sr., Williams SD , Einhorn LH. Klinefelter’s syndrome associated with mediastinal germ cell neoplasms. J Clin Oncol 1987; 5: 1290–1294. 3040921 10.1200/JCO.1987.5.8.1290

[11] Cushing B , Giller R , Cullen JW , Marina NM , Lauer SJ , Olson TA , et al. Randomized comparison of combination chemotherapy with etoposide, bleomycin, and either high-dose or standard-dose cisplatin in children and adolescents with high-risk malignant germ cell tumors: a pediatric intergroup study--Pediatric Oncology Group 9049 and Children’s Cancer Group 8882. J Clin Oncol 2004; 22: 2691–2700. 15226336 10.1200/JCO.2004.08.015

[12] Mann JR , Raafat F , Robinson K , Imeson J , Gornall P , Sokal M , et al. The United Kingdom Children’s Cancer Study Group’s second germ cell tumor study: carboplatin, etoposide, and bleomycin are effective treatment for children with malignant extracranial germ cell tumors, with acceptable toxicity. J Clin Oncol 2000; 18: 3809–3818. 11078494 10.1200/JCO.2000.18.22.3809

[13] Plant AS , Chi SN , Frazier L. Pediatric malignant germ cell tumors: a comparison of the neuro-oncology and solid tumor experience. Pediatr Blood Cancer 2016; 63: 2086–2095. 27554756 10.1002/pbc.26165

[14] Shaikh F , Murray MJ , Amatruda JF , Coleman N , Nicholson JC , Hale JP , et al. Paediatric extracranial germ-cell tumours. Lancet Oncol 2016; 17: e149–e162. 27300675 10.1016/S1470-2045(15)00545-8

[15] Nassir A. Testicular tumor at King Faisal Specialist Hospital and Research Centre - Jeddah, Kingdom of Saudi Arabia. UQU Medical Journal 2011; 2: 20–27.

[16] Williams LA , Frazier AL , Poynter JN. Survival differences by race/ethnicity among children and adolescents diagnosed with germ cell tumors. Int J Cancer 2020; 146: 2433–2441. 31304572 10.1002/ijc.32569 PMC6960364

[17] Mann JR , Raafat F , Robinson K , Imeson J , Gornall P , Phillips M , et al. UKCCSG’s germ cell tumour (GCT) studies: improving outcome for children with malignant extracranial non-gonadal tumours--carboplatin, etoposide, and bleomycin are effective and less toxic than previous regimens. United Kingdom Children’s Cancer Study Group. Med Pediatr Oncol 1998; 30: 217–227. 9473756 10.1002/(sici)1096-911x(199804)30:4<217::aid-mpo3>3.0.co;2-j

[18] Marina N , Fontanesi J , Kun L , Rao B , Jenkins JJ , Thompson EI , et al. Treatment of childhood germ cell tumors. Review of the St. Jude experience from 1979 to 1988. Cancer 1992; 70: 2568–2575. 1384951 10.1002/1097-0142(19921115)70:10<2568::aid-cncr2820701028>3.0.co;2-1

[19] Billmire D , Vinocur C , Rescorla F , Colombani P , Cushing B , Hawkins E , et al. Malignant retroperitoneal and abdominal germ cell tumors: an intergroup study. J Pediatr Surg 2003; 38: 315–318. 12632341 10.1053/jpsu.2003.50100

[20] İncesoy-Özdemir S , Ertem U , Şahin G , Bozkurt C , Yüksek N , Ören AC , et al. Clinical and epidemiological characteristics of children with germ cell tumors: a single center experience in a developing country. Turk J Pediatr 2017; 59: 410–417. 29624221 10.24953/turkjped.2017.04.007

[21] Kaatsch P , Häfner C , Calaminus G , Blettner M , Tulla M. Pediatric germ cell tumors from 1987 to 2011: incidence rates, time trends, and survival. Pediatrics 2015; 135: e136–143. 25489016 10.1542/peds.2014-1989

[22] Brodeur GM , Howarth CB , Pratt CB , Caces J , Hustu HO. Malignant germ cell tumors in 57 children and adolescents. Cancer 1981; 48: 1890–1898. 7284981 10.1002/1097-0142(19811015)48:8<1890::aid-cncr2820480830>3.0.co;2-d

[23] Poynter JN , Amatruda JF , Ross JA. Trends in incidence and survival of pediatric and adolescent patients with germ cell tumors in the United States, 1975 to 2006. Cancer 2010; 116: 4882–4891. 20597129 10.1002/cncr.25454 PMC3931133

[24] Fosså SD , Aass N , Winderen M , Börmer OP , Olsen DR. Long-term renal function after treatment for malignant germ-cell tumours. Ann Oncol 2002; 13: 222–228. 11885998 10.1093/annonc/mdf048

[25] Travis LB , Fosså SD , Schonfeld SJ , McMaster ML , Lynch CF , Storm H , et al. Second cancers among 40,576 testicular cancer patients: focus on long-term survivors. J Natl Cancer Inst 2005; 97: 1354–1365. 16174857 10.1093/jnci/dji278

[26] Haugnes HS , Aass N , Fosså SD , Dahl O , Brydøy M , Aasebø U , et al. Pulmonary function in long-term survivors of testicular cancer. J Clin Oncol 2009; 27: 2779–2786. 19414680 10.1200/JCO.2008.18.5181

[27] Fosså SD , Gilbert E , Dores GM , Chen J , McGlynn KA , Schonfeld S , et al. Noncancer causes of death in survivors of testicular cancer. J Natl Cancer Inst 2007; 99: 533–544. 17405998 10.1093/jnci/djk111

[28] Oeffinger KC , Mertens AC , Sklar CA , Kawashima T , Hudson MM , Meadows AT , et al. Chronic health conditions in adult survivors of childhood cancer. N Engl J Med 2006; 355: 1572–1582. 17035650 10.1056/NEJMsa060185

